# Associations between autism traits and family functioning over time in autistic and non-autistic children

**DOI:** 10.1177/13623613231151784

**Published:** 2023-02-08

**Authors:** Leontine W ten Hoopen, Pieter FA de Nijs, Geerte Slappendel, Jan van der Ende, Dennis Bastiaansen, Kirstin Greaves-Lord, Leona Hakkaart-van Roijen, Manon HJ Hillegers

**Affiliations:** 1 Erasmus MC Sophia Children’s Hospital, The Netherlands; 2 Erasmus School of Health Policy & Management, The Netherlands; 3 Yulius Mental Health, The Netherlands; 4 Lentis Mental Health, The Netherlands

**Keywords:** autism, caregivers, children, family functioning, longitudinal, parenting, traits

## Abstract

To explore associations between autism traits and family functioning over time, we studied longitudinal data of a mixed group of 168 clinically referred autistic and non-autistic children. Cross-lagged models showed a significant association between fewer autism traits at the diagnostic assessment and better family functioning 1 year later for the whole group, independently of children’s internalizing or externalizing behavior. When splitting the group into autistic children (58%) and non-autistic children (42%) based on an autism diagnosis, this association was only significant in the subgroup of non-autistic children with autism traits. We hypothesized that the needs of families experiencing difficulty understanding and adjusting to their children with autism traits, but no clinical autism diagnosis, might be unmet without the training or support facilities offered to families with autistic children. Although further research is needed to explore this association, clinicians may also consider supporting families of non-autistic children with autism traits to prevent family functioning problems. Because high autism trait levels in non-autistic children may be of a different origin than autism, for example, other neurodevelopmental or mental health problems, family training or support should be tailored to the child’s underlying difficulties.

**Lay Abstract:**

Little is known about family functioning over time when raising a child with autism traits, with or without a clinical autism diagnosis. Therefore, we asked caregivers—mostly parents—of a group of 168 children about the family functioning and the child’s emotional and behavioral characteristics, as well as autistic traits, twice with about 1 year in between. For numerous reasons, the children were referred to youth mental health care centers, including child and adolescent psychiatric services. Care as usual was offered after the diagnostic assessment if a clinical diagnosis was the assessment outcome. Caregivers reported less problematic family functioning in children with fewer autism traits over time. The child’s additional emotional or behavioral characteristics did not seem to influence this relation. Furthermore, we split the whole group into autistic children with a clinical autism diagnosis (58%) and non-autistic children with autism traits but without a clinical diagnosis (42%) to see whether we would find the same results in both groups. Surprisingly, the relation between family functioning and the level of a child’s autism traits only held for the subgroup of non-autistic children with autism traits. Thus, raising children with autism traits without a clinical diagnosis may affect family functioning over time. We think that families might have difficulty understanding and adjusting to the autism traits of their children but are lacking the support that is exclusively offered to families of children with a clinical autism diagnosis. We must be cautious because we do not know whether there is a causal relation. Although further research is needed to explore and learn to understand this result, clinicians might consider offering support to families of children with subthreshold autism to prevent problems in family functioning. Because high autism trait levels in non-autistic children may be of a different origin than autism, for example, other neurodevelopmental or mental health problems, family training or support should be tailored to the child’s underlying difficulties.

## Introduction

Autism is characterized by differences and difficulties in social communication and social responsiveness, as well as sensory, restricted, and repetitive behaviors (American Psychiatric Association [APA], 2013; Lord et al., 2022). Co-occurring emotional and behavioral problems are prevalent (Lai et al., 2019). Therefore, raising an autistic child might impact family life and function in search of understanding the child and adjusting to the individual needs (Volkmar et al., 2014). Caregivers, mostly parents, report a significantly lower quality of life (QoL) than the general population (Khanna et al., 2011; Kuhlthau et al., 2014). However, most caregivers of autistic children also indicate experiencing fulfillment from caring for their child (Hoefman et al., 2014; Ten Hoopen et al., 2020). Because of the continuous reciprocal interaction process between children and their families (Rodriguez et al., 2019), family functioning is an essential factor to consider in the child’s functioning and providing care to families with autistic children (Volkmar et al., 2014).

The heterogeneity of autism is known, with individual autism trait levels, strengths, problems, and needs varying over time (Lord et al., 2022). For a clinical diagnosis of autism,^1^ a significant impairment of the autism traits in essential areas of current functioning is required (APA, 2013). However, individuals with high autism trait levels but without sufficient impairment to meet autism diagnostic criteria might still show varying degrees of problems in social communication, social responsiveness, and repetitive behavior patterns. High autism trait levels might also be associated with other problems, such as problems in language use, flexible thinking, and behavior regulation (Sucksmith et al., 2011), and are not specifically related to autism (Lord & Bishop, 2021). Elevated autism traits are also present in children with other neurodevelopmental, such as language disorders, attention-deficit/hyperactivity disorder (ADHD), and other mental health conditions (Lord & Bishop, 2021). For example, Skuse et al. (2009) found functional impairment at school in a general population cohort of children. Kanne et al. (2009) found more psychosocial and psychiatric problems, such as increased anxiety and depression, in a general population group of non-autistic students with high levels of autism traits. Therefore, it is conceivable that non-autistic children with high levels of autism traits and their families might experience similar problems as autistic children and their families, although maybe to some lesser extent (Kanne et al. 2009).

Studies on the relationship between autism traits in children and family functioning are still scarce. Family functioning refers to the ability of cohesiveness, interaction, effective communication, decision-making and problem-solving, and getting along with each other, contributing to the development of the family and each family member (Epstein et al., 1983). There is some indication that autism or developmental disorder diagnoses in children are associated with impaired family functioning. Lee et al. (2008) found in a large United States cohort (*n* = 102.353) that parents of children with autism reported more negative effects on a self-composed scale with several dimensions of family functioning than parents of children with an attention deficit (hyperactivity) disorder (ADD/ADHD) or without a developmental disorder. Also, in an Israeli Bedouin Arab cross-sectional study with 400 mothers, raising a child with developmental disorders was related to lower family functioning on the McMasters Family Assessment Device (FAD) than raising a child without a developmental disorder (Manor-Binyamini, 2011). Finally, in an Australian cross-sectional study with 224 primary caregivers exploring predictive factors of family functioning in children with Down syndrome, co-occurring autism was associated with impaired family functioning on the FAD (Povee et al., 2012).

These findings raised two main research questions. First, might an association between autism and family functioning just hold for children with a clinical autism diagnosis, or does this association also apply to non-autistic children with high autism trait levels? Second, is there an association between autism trait levels and family functioning over time? We hypothesized that fewer autism traits in children could mean fewer difficulties for families in understanding the child and adjusting, potentially causing better family functioning, which may positively contribute to the individual child’s functioning (Karst & Van Hecke, 2012). Since previous studies were cross-sectional, they did not offer any temporal insight into this association. Herring et al. (2006) took a first step to explore the factors associated with family functioning on the FAD in autistic and non-autistic children with developmental delays, twice with 1 year in between. They found no significant influence of a clinical autism diagnosis, but emotional or behavioral problems predicted family functioning. Because most studies, including the study by Herring et al., (2006) only considered the categorical presence or absence of a clinical autism diagnosis, it offered no insight into the potential effect of continuous levels of autism traits on family functioning and vice versa over time. Importantly, assessing autism trait levels as a continuous measure in mixed or combined groups of autistic and non-autistic children might raise some metric questions (Murray et al., 2014). Often, for several research goals, instruments to measure autism traits in autistic children are also used to measure autism traits in non-autistic children. More and more, there is evidence that these two groups are separate categories, and autism traits might not be continuously distributed in the general population (Bölte et al., 2011; Kaat & Farmer, 2017; Murray et al., 2014). Therefore, we know little about to what extent an instrument measures autism traits the same way across autistic and non-autistic children. Under- or overestimating autism, or associations with autism, in one or both groups, is possible, so interpreting study results when using a continuous autism trait measure in a mixed autistic and non-autistic group should be done extra cautiously (Murray et al., 2014).

**Figure 1.**
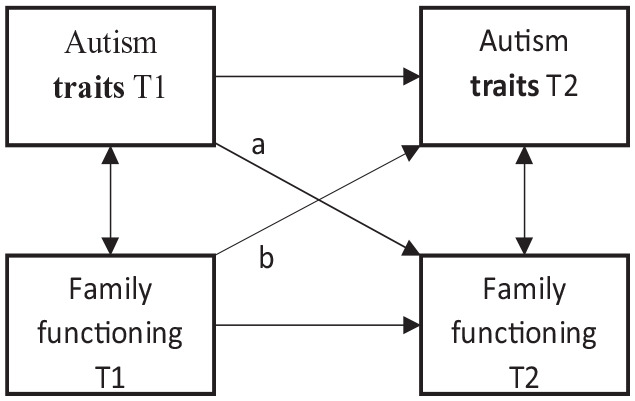
Regressive paths and cross-correlations in Model 1, excluding covariates.

Nevertheless, more insight into how autism traits and family functioning affect each other over time could offer new entry points for guidance, treatment, and outcome for children with autistic traits and their families, maintaining and improving their QoL. Therefore, in the current study, we explored longitudinal associations between family functioning and autism, continuously and categorically, over 1 year in a well-defined mixed clinically referred sample, including children with and without a clinical autism diagnosis (Duvekot et al., 2017). We hypothesized that these associations might best be described with a multidirectional, interactional model, as shown in Figure 1, with the level of autism traits affecting the family functioning over time and vice versa. We controlled for co-occurring internalizing and externalizing problems to ensure we could attribute revealed associations to autism traits rather than general emotional and behavioral problems (Herring et al., 2006). We also explored whether these associations differ for children with high autism trait levels who met the criteria for a clinical autism diagnosis versus children who did not meet the clinical autism diagnostic criteria. We expected that the multidirectional, interactional model would apply to both groups, although to some lesser extent to non-autistic children with high subclinical autism trait levels.

## Methods

### Sample description

Data were collected as part of the Social Spectrum Study, a prospective multicenter study of the heterogeneity of autism, exploring child, family, and societal factors associated with autism traits of clinically referred children in the southwest of the Netherlands (Duvekot et al., 2017). Before starting the data collection, the local medical ethics committee and the participating mental health care centers approved the study, including the study information and consent/assent procedure (MEC-2011-078). With the study information for the caregivers, we offered age-appropriate information for children younger than 6 years and children aged 6 years and older. On request, we provided additional information to the children and the caregivers. If they were willing to participate, we obtained written informed consent from the participating child’s caregivers or guardians (predominantly the parents) before all assessments.

For the study sample, we invited all children (age range 2–10 years) referred to one of six centers for child and adolescent mental health (CAMH) with high autism trait levels. With referrals for various developmental, emotional, and behavioral reasons, we selected the children with the parent-reported Social Responsiveness Scale, Second Edition (SRS-2; Constantino & Gruber, 2012) because of the screening accuracy (Duvekot et al., 2015). We identified the children with high autism trait levels (“screen-positive” children with a total score of ⩾75 on the SRS-2 vs “screen-negative” children with a total score of <75). We maximized the selection with an oversampling procedure (Duvekot et al., 2017). This first selection phase of the study took place from April 2011 till July 2012.

We invited the primary caregivers of 668 selected children to fill out questionnaires about child and family characteristics. Primary caregivers reported themselves as the caregiver most involved in parenting the child in the following categories: biological parent, foster parent, adoption parent, grandparent, step-parent, or otherwise. We did not include formal caregivers, such as physicians, psychologists, or therapists. Of all caregivers with completed questionnaires at T1 (*n* = 240), 70% completed questionnaires again after approximately 1 year at T2 (*n* = 168). The mean time between the two time points was 13 months (*SD* = 2.09). The participating caregivers were mostly mothers at T1 (95%) and T2 (91%). We found that caregivers who did not live together with a partner or had a child of non-Dutch ethnicity were less likely to participate in the follow-up (Duvekot et al., 2017).

### Community involvement

No members of the autism community were involved in the original study design, methodology, and conduct of the research. However, to test whether the used terminology was appropriate and the questionnaires were user-friendly, we asked some non-participating caregivers of children to fill out the whole set. Based on their feedback, we made minor text, order, or layout adjustments. Previous results of the Social Spectrum Study were disseminated to the participating children, caregivers, and CAMH via periodic newsletters. The authors will discuss the study results of the current study with members of the public via future conference presentations and posters.

### Instruments

#### Child autism traits

The caregiver reported the autism traits of the child at T1 and T2 on the 65 items of the *Social Responsiveness Scale*, Second Edition (*SRS-2*; Constantino & Gruber, 2012). We used the SRS-2 because of the ability to differentiate between autism and other mental health conditions in high-risk groups, such as clinically referred children (Constantino & Gruber, 2005). Items about social communication, interaction, and restricted and repetitive behavior were scored on the 4-point Likert-type scale from 0 (*not true*) to 3 (*almost always true*), with total scores ranging from 0 to 195. Higher scores were indicative of more autism traits. We used the largely similar pre-school version for the children in the age range <4 years (*n* = 6, 4%) and the school-age version for children in the age range of 4–10 years (*n* = 162, 96%), with only age-appropriate wording adaptions in 10 items without adapting the content. In the present study, we chose the total raw cut-off score of 75 on the parent-reported SRS to screen for autism, to differentiate between children with autism and children with other mental health conditions with a sensitivity of 0.85 and a specificity of 0.75 (Constantino & Gruber, 2005). The total raw score was recommended for research goals, with a threshold of 75 or higher used as a cut-off for a high likelihood of autism (Constantino & Gruber, 2005). The good psychometric properties of the Dutch translation (Roeyers et al., 2011) were previously established by the good reliability and validity in the Social Spectrum Study (Duvekot et al., 2015). We also found a high internal consistency (*α* = 0.96).

#### Family functioning

Family functioning at T1 and T2 was assessed by the caregiver using the 12 items of the General Functioning scale of the McMasters FAD (Epstein et al., 1983). Statements such as “Planning family activities is difficult because we misunderstand each other” and “In time of crisis we can turn to each other for support” were scored on the 4-point Likert-type scale from 1 (*strongly disagree*) to 4 (*strongly agree*). Half of the scores needed recoding because of reverse formulations, with a higher total score indicating more family problems (or less family functioning). The General Functioning Subscale of the FAD has shown good reliability and validity as a stand-alone screener (Byles et al., 1988). In the current study, the internal consistency was sufficient (*α* = 0.87).

#### Child internalizing and externalizing problems

The caregiver reported the child’s internalizing and externalizing problems on the Child Behavior Checklist (CBCL; Achenbach & Rescorla, 2000, 2001) at the start of the selection procedure and T2. Items about emotional and behavioral problems were scored on the 3-point Likert-type scale from 0 (*not true*) to 2 (*very true or often true*), with higher scores meaning more child problems. Dependent on the child’s age, caregivers filled out the CBCL 1.5–5 (*n* = 47; 30.3%) or the CBCL 6–18 (*n* = 108; 69.7%). We used *T*-scores of total internalizing and externalizing problems for the analyses because of the different item numbers of the internalizing and externalizing scales. Both versions of the CBCL have good validity and reliability (Achenbach & Rescorla, 2000, 2001). In our study, the internal consistency was sufficient to good (CBCL 1.5–5: α = 0.88; CBCL 6–18: α = 0.94). Exploring potential selection in the group of 13 children without a CBCL versus the group of 155 children with a CBCL, we found no significant differences in FAD total score, age, clinical autism diagnosis, Autism Diagnostic Observation Schedule (ADOS) classification, and Developmental Dimensional and Diagnostic Interview (3Di) classification. However, caregivers reported significantly fewer autism traits on the SRS-2 for the children with a completed CBCL compared to the children without a completed CBCL (*p* = 0.017).

#### Clinical autism diagnosis

For a clinical diagnosis of autism versus non-autism of the child, we used the best-estimate clinical diagnosis (BEC diagnosis). In BEC diagnoses, standardized scores or classifications on gold standard diagnostic measures are combined with clinical expertise (Lord, Petkova, et al., 2012). In our study, we based the BEC diagnosis on results of the Autism Diagnostic Observation Schedule (ADOS; Lord, Rutter, et al., 2012), the Developmental Dimensional and Diagnostic Interview (3Di; Santosh et al., 2009; Slappendel et al., 2016), and the clinical judgment according to *Diagnostic and Statistical Manual of Mental Disorders* (4th ed.; text rev.; *DSM*-IV-TR; Duvekot et al., 2017). Experienced clinicians of the research team, who performed the assessments, made these research diagnoses, apart from the standard clinical diagnostic service. BEC diagnoses were available only for those children participating in both diagnostic assessments (ADOS and 3Di) as part of the Social Spectrum Study, leading to complete data for 132 cases. Of these 132 children, 76 (58%) were classified as having autism (autistic children) and 56 (42%) as non-autism (non-autistic children). We found no differences between the autistic and non-autistic children in ethnic background, full-scale IQ, number of siblings, intactness of household, and sex and education level of the caregiver. However, autistic children were more often male (82% vs 58%), younger (8.33 years (*SD* = 2.08) vs 9.57 years (*SD* = 2.34)), with younger caregivers (37.32 years (*SD* = 5.03) vs 39.87 (*SD* = 4.96)). Children without available BEC diagnosis (*n* = 36) were included in the primary models for the entire group but not in the subsequent analyses, splitting into subgroups of autistic versus non-autistic children. We found no significant differences between included and excluded children in the scores on the SRS-2, FAD, CBCL internalizing or externalizing behavior, age, or classification by ADOS or 3Di.

#### Covariates

Several variables were entered into the models as covariates to rule out the effects of potential confounding factors. First, low income and poverty are inversely related to family functioning (Banovcinova et al., 2014). With lacking information on family income, we used *maternal education level*, as reported by the caregiver, as a proxy for socio-economic standard, classified as low, medium, and high educational levels. Second, family intactness seems to be associated with better family functioning (Freistadt & Strohschein, 2013; Shek et al., 2014). *Family intactness* was coded based on a question asking caregivers to report their child’s living situation, that is, with intact biological or adoptive family, single biological or adoptive parent, biological or adoptive parent with a new partner, or other, such as foster care or in-patient mental health care. Also, we included the *child’s age, sex, and number of siblings* as covariates.

### Statistical analyses

Because of the oversampling design in the original study, we weighted the final sample data (*n* = 168) to represent the original total eligible sample (see Duvekot et al., 2015 for the weighing procedure). Therefore, the probability that each child would be selected from the total screened sample was calculated, with a 100% probability of screen-positive children and a 29% probability on average of screen-negative children. Each case was weighted with the inverse of the probability of inclusion in the final dataset, leading to data of 121 screen-positive children having a weight of 3.65 and data of 47 screen-negative children weighing 1 in the analyses. With this weighting procedure, we compensated for the underrepresentation of screen-negative children in the study sample.

All analyses were performed with the weighted data. We analyzed the study sample with SPSS version 24.0. Cross-lagged models were fit using Mplus 8.5 (www.statmodel.com). All models included child age and sex, family intactness, number of siblings, and maternal education level as covariates, regressed on SRS-2 and FAD scores at T2. Maximum likelihood estimation (MLR) with robust estimation of standard errors was used in all tested models. We determined the significance of the test results at an alpha level of 0.05.

First, we ran descriptive statistics (i.e. mean and standard deviation, or percentages) for demographic variables and SRS-2, FAD, and CBCL at two time points. Next, auto-regressive cross-lagged models were fit for the entire group (*n* = 168). We excluded children without FAD data at T1 (*n* = 13). Mplus uses full-information maximum likelihood (FIML) estimation to account for missing data.

In step 1 of the model fitting, we tested a baseline model with only auto-regressive paths (horizontal paths in Figure 1) and concurrent correlations at T1 and T2 (vertical paths in Figure 1). Then, we added cross-lagged paths one by one to compare the added influence of each path. In step 2, the cross-lagged path from SRS-2 at T1 to FAD at T2 (path a in Figure 1) was added to this model, as this is the influence that has the most support in previous literature (Herring et al., 2006). In step 3, the second cross-lagged path was added, from FAD at T1 to SRS-2 at T2 (path b in Figure 1). After deciding on a final model based on the fit indices, we expanded this model with the child’s co-occurring internalizing and externalizing problems to explore whether the influences of autism traits would lose their significance. In that case, these problems could be responsible for the difference in family functioning rather than autism traits per se. Internalizing and externalizing problems were added to the regression models for autism traits and family functioning at both T1 and T2.

To further explore whether the association between autistic traits and family functioning was specific to children with a BEC diagnosis of autism or also existed in children with high subclinical autism trait levels, the final model without internalizing and externalizing problems was rerun with a group split by BEC diagnosis (autistic vs non-autistic). For the split analysis, we took two steps: first, a fully constrained model—that is, all parameters were constrained to be identical between the two groups—was run as a baseline model. Then we removed the constraints for the association between autism traits at T1 and family functioning at T2 to test whether this would improve the model fit relative to the fully constrained model. Since BEC diagnosis was not available for all children, this analysis included a total of 132 children before weighting, of whom 76 received an autism diagnosis (autistic children) and 56 did not receive an autism diagnosis (non-autistic children).

We evaluated the model fit to the data with root mean square error of approximation (RMSEA), comparative fit index (CFI), and the standardized root mean residual (SRMR) (Byrne (2012). The model fit was rated as good with RMSEA < 0.05, CFI ⩾ 0.95, and SRMR < 0.05 (Hu & Bentler, 1998). We compared the nested models and multiple groups with chi-square tests.

## Results

Table 1 shows the characteristics of the weighted study sample. The children were mostly male (68%), of Dutch origin (89%), on average 9.07 years (*SD* = 2.26) old at T1; 46% of the weighted sample had a BEC diagnosis of autism (autistic children). Most children came from intact biological or adoptive families (75%). The caregivers were primarily female (97%), the biological mother (92%), on average 38.40 years (*SD* = 5.10) old, and 26% of a high educational level at T1.

**Table 1.** Study sample characteristics and descriptives weighted sample (*n* = 290).

	Characteristics	*M* (*SD*)/%
Child characteristics	Sex (% boys)	67.8
	Age (years)	9.07 (2.26)
	Ethnicity (% Dutch)^a^	89.1
	Full-scale IQ	98.78 (16.78)
	SRS-2	
	- Total score at T0	69.72 (28.99)
	- Total score at T1	69.63 (32.50)
	- Total score at T2	65.46 (32.16)
	Autism diagnosis (%)	
	- ADOS classification	40.6
	- Best-estimate clinical diagnosis	45.9
	CBCL	
	- Total internalizing *T*-score at T0	63.33 (10.13)
	- Total internalizing *T*-score at T2	59.04 (11.40)
	- Total externalizing *T*-score at T0	64.18 (10.97)
	- Total externalizing *T*-score at T2	55.90 (11.56)
Caregiver characteristics	Sex (% female)	96.9
	Age (years)	38.40 (5.10)
	Biological parent of the child (%)	95.1
	Biological mother of the child (%)	92.4
	Education level (%)	
	- Low	25.3
	- Medium	46.5
	- High	26.7
Family characteristics	Total number of siblings	1.44 (1.20)
	Intactness household (%)^b^	75.3
	FAD	
	- Total score at T1	20.58 (4.78)
	- Total score at T2	19.61 (4.96)

M: mean; *SD*: standard deviation; SRS-2: Social Responsiveness Scale, Second Edition; FAD: McMasters Family Assessment Device; IQ Intelligence Quotient; ADOS: Autism Diagnostic Observation Schedule; CBCL: Child Behavior Checklist; INT: internalizing; EXT: externalizing; T0: at start of the selection procedure; T1: first assessment; T2: second assessment.

a Ethnicity was classified as “Dutch” if both parents were born in the Netherlands.

b Intactness household is defined as a living situation with both biological or adoptive parents.

Table 2 shows the model fit for the initial, full group models. The cross-lagged path from autism traits at T1 to family functioning at T2 (path a in Model 1, Figure 1) was significant in both the full (*β* = 0.175, *p* = 0.018) and partial cross-lagged model (*β* = 0.177, *p* = 0.016), meaning fewer autism traits of the child were associated with fewer problems in family functioning over time. As shown in Table 2, the model with the partial cross-lagged path 1a improved the baseline model significantly (*Δχ*^2^ (1) = 5.5, *p* = 0.019), although the fit to the data of this model was less than adequate (*χ*^2^ (11) = 31.8, *p* < 0.01; CFI = 0.89; RMSEA = 0.11; SRMR = 0.09). The second cross-lagged path from family functioning at T1 to autism traits at T2 (path b in Model 1, Figure 1) was not significant (*β* = 0.023, *p* = 0.686), and including path b did not improve model fit compared with including only path a (*Δχ*^2^ (1) = 0.05, *p* = 0.82). This finding indicated no association between less problematic family functioning at T1 and fewer autism traits in children at T2.

**Table 2.** Fit indices for tested models.

Model	*χ*^2^ (*df*)	RMSEA (90% CI)	CFI	SRMR	*Δχ*^2^ (*df*)
1. Parallel	37.6 (12)	0.11 (0.07–0.15)	0.87	0.10	–
1a. Partial cross-lagged	31.8 (11)	0.11 (0.06–0.15)	0.89	0.09	5.5* (1)
1ab. Full cross-lagged	32.3 (10)	0.12 (0.07–0.16)	0.88	0.09	0.05 (1)
2. Partial cross-lagged with co-occurring symptoms	59.1 (21)	0.10 (0.07–0.14)	0.86	0.08	–

CI: confidence interval; *df* degrees of freedom (nonsignificant χ^2^ values indicate adequate fit and are indicated with *); RMSEA: root mean square error of approximation (<0.08 suggests adequate fit, <0.05 suggests good fit); CFI: comparative fit index (>0.90 suggests adequate fit, >0.95 suggests good fit); SRMR: standardized root mean residual (<0.08 suggests adequate fit, <0.05 suggests good fit); Δχ^2^: chi-square difference between models.

* Indicates adequate fit and a significant improvement relative to the previous model at *p* < 0.05.

Note that Model 2 contains extra variables and therefore cannot be directly compared with the other models.

**Figure 2.**
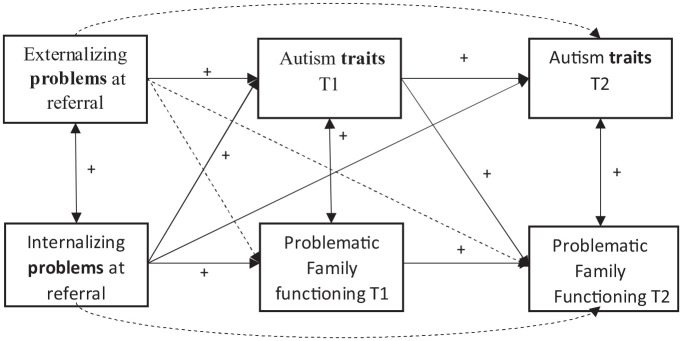
Regressive paths and cross-correlations in Model 2, including co-occurring problems at referral. Dotted arrows indicate nonsignificant paths in the model without covariates.

To explore whether co-occurring problems might influence the association between autism traits of the child on T1 and family functioning on T2, we added co-occurring total internalizing and externalizing problems of the child to the final model including only path a. The expanded model with total internalizing and externalizing problems (Model 2, Figure 2) did not change the significant association between autism traits at T1 and family functioning at T2 (*β* = 0.235, *p* = 0.004). Internalizing and externalizing problems did not contribute significantly to autism traits at T2 (internalizing: *β* = 0.093, *p* = 0.121) or family functioning at T2 (internalizing: *β* = −0.125, *p* = 0.139; externalizing: *β* = 0.023, *p* = 0.777), except for a significant association between externalizing problems and autism traits (*β* = 0.125, *p* = 0.049) at T2. The paths in both models did not change when covariates were removed. Again, internalizing and externalizing problems were not significantly contributing to autism traits at T2 (externalizing: *β* = 0.098, *p* = 0.116) or family functioning at T2 (internalizing: *β* = −0.136, *p* = 0.108; externalizing: *β* = 0.056, *p* = 0.459), except for a significant association between internalizing problems and autism traits (*β* = 0.125, *p* = 0.049) at T2. Table 2 also shows that the fit of Model 2 to the data was less adequate (*χ*^2^ (21) = 59.1, *p* < 0.00001; CFI = 0.86; RMSEA = 0.10; SRMR = 0.08). Due to differences in the variables included in the different models, we could not directly compare the fit between Models 1 and 2. However, both models agreed on a significant association between T1 autism traits and T2 family functioning but no association between T1 family functioning and T2 autism traits. Thus, adding co-occurring internalizing and externalizing problems to the model did not appear to change these associations.

Table 3 shows model fit indices for the models split by the autism diagnosis, with the group of autistic children versus the group of non-autistic children. The baseline model constrained all parameters to be equal, whereas Model 1 released the path from autism traits at T1 to family functioning at T2 to differ between groups. The change in chi-square (*Δχ*^2^ (1) = 29.10, *p* < 0.0001) indicated that the association between autism traits at T1 and family functioning at T2 is different between both groups. The exploration remained significant for the non-autistic group (*β* = 0.375, *p* < 0.0001), but not for the autistic group (*β* = −0.067, *p* = 0.588), meaning that fewer autism traits of the child were associated with less problematic family functioning in 1 year time in solely the group of non-autistic children with high subclinical autism trait levels, but not in the group of autistic children.

**Table 3.** Fit indices for tested split models.

Model	*χ*^2^ (*df*)	RMSEA (90% CI)	CFI	SRMR	Δ*χ*^2^ (*df*)
Baseline model	48.1 (25)	0.12 (0.07–0.17)	0.85	0.15	–
1. Freed SRS-2 T1 ⩾ FAD T2	37.7 (24)	0.09 (0.02–0.15)	0.91*	0.12	29.10 (1)** *p* < 0.0001

CI: confidence interval; SRS-2: Social Responsiveness Scale; Second Edition; FAD: McMasters Family Assessment Device; *df*: degrees of freedom (non-significant χ^2^ values indicate adequate fit and are indicated with *); RMSEA: root mean square error of approximation (<0.08 suggests adequate fit, <0.05 suggests good fit); CFI: Comparative Fit Index (>0.90 suggests adequate fit, >0.95 suggests good fit); SRMR: standardized root mean residual (<0.08 suggests adequate fit, <0.05 suggests good fit); *Δχ*^2^: chi-square difference between models.

* Indicates a significant improvement relative to the previous model at *p* < 0.05 (indicates adequate fit).

** *p* < 0.01 (indicates good fit).

Note that Model 2 contains extra variables and therefore cannot be directly compared with the other models.

## Discussion

This study was the first to explore longitudinal associations between autism traits and family functioning over 1 year in a well-defined clinically referred mixed sample of autistic and non-autistic children aged 2–10 years with high autism trait levels. We tested (1) the reciprocal longitudinal associations between child autism traits and family functioning, (2) whether the inclusion of co-occurring internalizing and externalizing problems changed these associations, and (3) explored whether associations were specific to children with or without a clinical autism diagnosis. Our analyses showed a longitudinal association between fewer autism traits and better family functioning approximately 1 year later, which concurred with a previous study (Herring et al., 2006). The found association was not changed by adding co-occurring internalizing and externalizing problems. Moreover, further explorative analysis revealed that autism traits were associated with family functioning over time only in children with high subclinical autism trait levels who did not meet the criteria for a clinical autism diagnosis. Thus, fewer autism traits appear to be associated with better family functioning over time only in children with high subclinical autism trait levels. We highlight some results in more detail to provide a perspective on the longitudinal association between family functioning and autism traits.

Our models suggest a unidirectional longitudinal association between autism traits at the first assessment and family functioning at the second assessment, approximately 1 year later, which concord with a previous study (Herring et al., 2006). Although the continuous reciprocal interaction between child and family functioning seems logical and is often emphasized (Lord et al., 2022; Rodriguez et al., 2019), and although we hypothesized a multidirectional model, our analyses did not support this model. We must be cautious in interpreting the results because of differences in sample size and age range, including rather well-functioning children with variable autism trait levels, and last but not least, less adequate model fit. Also, we only found a temporal association; potential causality between autism trait levels in children and family functioning over time needs further investigation.

In contrast to the study results of Herring et al. (2006), based on our data, we could not explain the association between autism traits and family functioning by co-occurring internalizing or externalizing problems. Although previous studies found stability of the impact of emotional and behavioral problems in, respectively, toddlers and youngsters with autism on the caregivers (Herring et al. 2006; Lecavalier et al., 2006), there are many differences in age range, problem levels, and used instruments. Our study sample consisted of rather well-functioning children with little developmental delay (Table 1), including the children with high subclinical autism trait levels, which might explain the differences.

Importantly, in our explorative analyses, we found the association between fewer autism traits in children and better family functioning, approximately 1 year later, only to hold for the non-autistic children with high autism traits, not for the autistic children. This result may initially seem counterintuitive, given that one would expect a more significant positive effect on family functioning from less impairment in children, as we hypothesized. However, families with an autistic child may already have received a supporting treatment plan and guidance in understanding and adjusting to the child’s autism traits during the measurement interval. Families of non-autistic children with high autism trait levels might not have provided any services, potentially leaving them searching for parenting strategies to overcome potential difficulties. Also, high autism trait levels in non-autistic children may be related to other neurodevelopmental or mental health problems than autism, with family training or support maybe not tailored enough to the child’s underlying difficulties. In addition, a clinical autism diagnosis might mean quite a difference for families in their view on experienced problems, instead of worrying about the cause of the problems and limitations of the child. Flanders et al. (2007) found higher healthcare costs in the prediagnostic phase in autistic children in a North-American healthcare consumption study, possibly reflecting extensive searching for an explanation or diagnosis for the child’s problems. Our analyses did not allow us to test these explanations of our findings. Therefore, we speculate that families with an autistic child may have access to more guidance and treatment planning in understanding and adjusting their interaction style and expectations to the needs and abilities of the child. Such adaptations may have resulted in fewer family problems due to the child’s functioning. In contrast, families of non-autistic children with high autism trait levels may experience more or longer difficulty understanding, accepting, and adjusting to their child’s functioning. Therefore, in concordance with our findings, Lord et al. (2022) recommended the assessment and support to be more integrated and personalized in clinical practice, focusing more on individual and family needs, and thus including a broader, more heterogeneous group of children with autism traits.

### Strengths and limitations

This study has several strengths. First, the longitudinal study design (Duvekot et al., 2017), including an in-depth diagnostic assessment and reassessment about 1 year later, provided information about the evolution of autism traits and family functioning in a well-defined group of clinically referred autistic children, but also non-autistic children with high autism traits. Second, we incorporated the continuous measurement of autism trait levels (SRS-2) as well as the categorically assessed variable of an autism diagnosis (BEC diagnosis) to overcome the limitations of one or the other (Herring et al., 2006). Third, by using this design, we could also include children with subclinical autism traits, potentially gathering more information about this somewhat understudied group. Finally, we used multiple informants (primary caregivers and clinician-experts) for the BEC diagnosis of the children to add information to standardized one-informant instruments (Lord, Petkova, et al., 2012).

However, we should interpret the results of our study cautiously, considering the several important limitations. First, although the best model with the path from autism trait levels at T1 to family functioning at T2 was a significant improvement of the baseline model, with robust outcomes in both models, the fit of the models to our data was less adequate (see Tables 2 and 3 for fit indices). Because of the study’s exploratory nature in a relatively small sample of cases, we still think the findings are clinically relevant. These associations should be further investigated and replicated in larger representative samples of clinically referred children.

Second, as indicated before, we must be aware that high subclinical autism traits may not always be specifically related to autism. Elevated autism trait scores may also be found in children with emotional and behavioral problems, and neurodevelopmental or mental health conditions (Lord & Bishop, 2021). In autism trait measurement, not all items are specific to autism and are not as such recognized by caregivers. At a behavioral level, there are superficial overlaps between autism, and neurodevelopmental and mental health conditions, although these characteristics have different causes. For example, sensory processing differences, often reported as autism traits, are associated with a broad spectrum of mental health problems (Van den Boogert et al., 2022). Thus, children’s high subclinical autism trait levels may be related to various mental health problems or conditions, a milder autism presentation, or both. Current autism measures, such as the SRS-2, seem unable to distinguish based on the origin of the traits. In the present study, we could not establish whether the autism traits were related to other neurodevelopmental or mental health conditions because of the lack of comprehensive patient file information for the entire group of children. In studying autism traits, any existing neurodevelopmental or mental health problems must be considered as much as possible. Also, we have to be aware that some non-autistic children with high autism trait levels may later receive a clinical autism diagnosis, a neurodevelopmental or mental health condition because of more functional impairments, less social or cognitive compensating or camouflaging (Lord & Bishop, 2021). In further longitudinal research, it would be interesting to find out which non-autistic children with high autism trait levels are due to make this transition and to investigate whether interventions could help to prevent this.

Third, we must be cautious in interpreting autism trait measurements for several reasons. Using a single measure for multiple purposes and across different groups in autism research, we should consider metric aspects such as measurement equivalence, metric invariance, and scalar invariance in interpreting study results (Kaat & Farmer, 2017; Murray et al., 2014). There is growing evidence that the group of autistic children might best be characterized as distinct from the group of non-autistic children with high autism trait levels (Frazier et al., 2010, 2012; Lord & Bishop, 2021). Therefore, we have to question the SRS-2 as a continuous measure in the whole mixed group of autistic and non-autistic children in our study. Consequently, the risk of systematically over- or underestimating the autism trait scores in one or both groups is present (Murray et al., 2014). In addition, although items about restricted and repetitive behavior (RRB) are part of the SRS-2, most are about social responsiveness and communication (Constantino & Gruber, 2012). Specific RRB measurements may be recommended to sufficiently cover this domain of autism in clinical care and research. The specificity of the SRS-2 is often low in groups of children referred for autism diagnostic assessment (Aldridge et al., 2012). However, in our study, the specificity of the SRS-2 was 0.75 (Duvekot et al., 2015). Despite the disadvantages, we used the SRS-2 to investigate the association between autism traits and family functioning because of better suitability in clinical samples than some other measures (Bölte et al., 2011). In future research, associations with autism traits would preferably be investigated in both groups (autistic vs non-autistic children) separately or, with enhanced caution, that is, with the metric aspects considered, in a mixed group.

Fourth, we could not include information about the caregiver’s stress, mental and physical health problems, or sibling functioning problems in the study models. Autism traits of a child may affect the entire family, including caregivers and siblings. Caregivers of autistic children report more stress and mental health problems (Hastings et al., 2005; McStay, Dissanayake, et al., 2014; McStay, Trembath, & Dissanayake, 2014) because of the severity of child problems, care needs, low support, and concerns about the siblings’ functioning. Next to the high penetrance of autism in families (Constantino & Todd, 2003; Volkmar et al., 2014), Duvekot et al. (2016) found a genetic predisposition of autism and internalizing problems in caregivers of a child with autism traits. These caregiver and caregiving characteristics may also increase challenges in raising children with autism traits and experiences family functioning independent of a clinical autism diagnosis. Therefore, they are essential in providing parent guidance and support, but also in considering the results of the current and future studies.

Fifth, we have to consider the study sample’s representativeness concerning the results’ generalizability. As mentioned before, the selected 168 children were referred to CAMH centers for various reasons but were rather well-functioning compared with children in other study samples (Herring et al., 2006). Most children came from intact families, lived with their biological parents, and did not have a developmental delay (Table 1). The mean *T*-scores for the children’s co-occurring emotional and behavioral problems indicated they were at risk, but not above the cut-off, for clinical symptoms (Achenbach & Rescorla, 2000, 2001). Also, 26% of the primary caregivers reported themselves as highly educated. Despite the clinically relevant associations, we cannot generalize the findings to the general population because we based them on measurements in a referred sample with relatively high-functioning families.

Finally, information about provided services, treatment, or support is lacking in the interval between the two assessments. Care as usual was offered, which could encompass no treatment for children without a clinical autism diagnosis to complete treatment and support for children with a clinical autism diagnosis according to international standards (Volkmar et al., 2014). Therefore, we could not test whether families with an autistic child received better treatment planning and support than those with a non-autistic child with high autism trait levels, and whether this affected family functioning (Factor et al., 2019). For preventing or improving family functioning in clinical practice, information about provided treatment and support is essential in further research.

### Clinical implications and future research

The association between child autism traits and family functioning over time, especially in children with high subclinical autism trait levels, could be very relevant for clinical support and treatment guidelines. Naturally, replication in larger, more representative study samples or separated samples of autistic and non-autistic children, with a longitudinal study design, including more child clinical diagnosis information, family characteristics, and intervention information, is needed to overcome the limitations of this study. To improve family functioning and QoL, we should reconsider offering more education, support, and even treatment to families of non-autistic children with high subclinical autism trait levels instead of only providing this to families of autistic children. Because high autism trait levels in non-autistic children may be related to other neurodevelopmental or mental health problems than autism, family training or support should be foremost tailored to the child’s underlying difficulties. Such an adjustment in guidelines could imply a paradigm shift in healthcare systems that only cover diagnosis-based treatment and guidance. Following the recommendations by Lord et al. (2022), focusing on the necessary individual and familial education, support, guidance, and treatment in children with developmental, emotional, and behavioral problems might be more effective in the long run than a solely diagnosis-oriented approach.

## Conclusion

The associations between children’s autism, internalizing and externalizing problems, diagnoses, and family functioning are complex. Our results indicated an association between autism traits in children and family functioning over approximately 1 year. In our study, co-occurring internalizing and externalizing problems could not explain this association. This finding mainly concerned non-autistic children with high autism trait levels. Given that these findings resulted from exploratory analyses in a relatively small subgroup and high autism trait levels are not exclusively related to autism, further research is needed to determine how diagnostic status might moderate the impact of autism traits on family functioning. Given the results of our study, clinicians may consider identifying emerging family problems and providing support to families in dealing with high subclinical autism traits in non-autistic children, specifically tailored to the child’s underlying difficulties.

## Notes

1. While the diagnostic category of the *Diagnostic and Statistical Manual of Mental Disorders* (*DSM*) nowadays is “Autism Spectrum Disorder,” the term “autism” will be used to align with community preferences.
